# The Impact of the COVID-19 Pandemic on the Unpredictable Dynamics of the Cryptocurrency Market

**DOI:** 10.3390/e23091234

**Published:** 2021-09-20

**Authors:** Kyungwon Kim, Minhyuk Lee

**Affiliations:** 1 Division of International Trade, Incheon National University, 119 Academy-ro, Yeonsu-gu, Incheon 22012, Korea; kk@inu.ac.kr; 2 Department of Business Administration, Pusan National University, Busan 46241, Korea

**Keywords:** cryptocurrency, unpredictability, COVID-19, approximate entropy, sample entropy, Lempel-Ziv complexity

## Abstract

The global economy is under great shock again in 2020 due to the COVID-19 pandemic; it has not been long since the global financial crisis in 2008. Therefore, we investigate the evolution of the complexity of the cryptocurrency market and analyze the characteristics from the past bull market in 2017 to the present the COVID-19 pandemic. To confirm the evolutionary complexity of the cryptocurrency market, three general complexity analyses based on nonlinear measures were used: approximate entropy (ApEn), sample entropy (SampEn), and Lempel-Ziv complexity (LZ). We analyzed the market complexity/unpredictability for 43 cryptocurrency prices that have been trading until recently. In addition, three non-parametric tests suitable for non-normal distribution comparison were used to cross-check quantitatively. Finally, using the sliding time window analysis, we observed the change in the complexity of the cryptocurrency market according to events such as the COVID-19 pandemic and vaccination. This study is the first to confirm the complexity/unpredictability of the cryptocurrency market from the bull market to the COVID-19 pandemic outbreak. We find that ApEn, SampEn, and LZ complexity metrics of all markets could not generalize the COVID-19 effect of the complexity due to different patterns. However, market unpredictability is increasing by the ongoing health crisis.

## 1. Introduction

After the financial crisis that put the global economy into a panic in 2008, many studies have established a system to predict a crisis in advance by using big data and artificial intelligence (AI) [1,2,3]. The financial markets seems to have stabilized, but the World Health Organization (WHO) declared COVID-19 as a pandemic in 2020. The global economy is once again in panic; this follows the global financial crisis in 2008. Consumer consumption has changed, and the types and quantities of products have also changed. Many companies are trying to escape from the panic by the changes in business and marketing, but they are still unable to recover their business or reduce their financial burden easily. In addition, the unemployment rate and the COVID-19 incidence rate are still not improving. As the liquidity of capital decreases in many ways due to these changes, there is also a phenomenon of seeking new investment destinations. Recent studies show that the effect of the recently developed COVID-19 vaccine will improve real assets and financial assets [4,5,6]. The cryptocurrency market also shows a sharp decline due to COVID-19 after booming in 2017. The cryptocurrency market is now a new financial market that needs a lot of research, including research on the impact of COVID-19. However, there has not been much research on predicting the future of the cryptocurrency market or measuring its impact on COVID-19. Therefore, from an investment perspective, we aim to examine the evolution of the complexity of the cryptocurrency market and analyze the characteristics of the market from the past bull market to the present COVID-19 pandemic outbreak to help policymakers and decision-makers ensure future stability.

There are studies about the cryptocurrency market that Böhme et al. [7] examine different aspects of Bitcoin (economics, technology, and governance), and Bariviera and Merediz-Solà [8] and Corbet et al. [9] conduct a meta-analysis about the cryptocurrencies. There are also existing studies on the cryptocurrency market checking its efficiency [10,11] and its fractality [12,13,14,15,16,17,18]. Using information theory, a study of comparing the price movement of major cryptocurrencies and measuring informational efficiency was conducted [19,20]. Goodell and Goutte [21] studies the co-movement of bitcoin and COVID-19. Additionally, a multi-scale bi-dimensional approach is studied for analyzing complex time-series [22]. Others investigate the market entropy [23,24,25,26,27] but concentrate on the short-term patterns. This study is the first to understand the mid-term to long-term social phenomena from the bull of cryptocurrency in 2017 to the current COVID-19 pandemic. In addition, it has been over 10 years since the cryptocurrency market started, and many cryptocurrencies have been newly developed and disappeared due to various issues. Therefore, analyzing all the cryptocurrency markets that are being traded stably so far can provide quantitative detailed market information without hastily generalizing all markets, including comparison tests in accordance with the market caps.

To investigate the evolutionary complexity, we conduct three general complexity analyses on the basis of nonlinear measures. First, Pincus [28] introduced Approximate Entropy (ApEn) which is suitable for measuring sequential and temporal regularity/irregularity of time series based on the existence of patterns in 1991. ApEn can represent the probability of a pattern that has not appeared so far [29]. Therefore, ApEn is one of the most popular metrics to estimate complexity and regularity. However, ApEn has bias, consistency, and dependence on the sample length because ApEn counts each sequence as matching itself [30]. To overcome the disadvantages of ApEn, Richman and Moorman [30] proposed the Sample Entropy (SampEn) in 2000 which does not count self-matches, so that SampEn is a more consistent and robust estimator than ApEn. SampEn is independent of the sample length. Therefore, we choose SampEn as the second complexity measurement methodology for comparing and analyzing the result values of ApEn for cross-checks and look at the complexity of time series data in various views. Finally, Lempel-Ziv complexity (LZ) [31] measures whether a new non-repetitive pattern exists in a time sequence. These metrics can easily determine the nonlinear and non-stationary sequence without any special prior distributional assumptions. The approximate entropy (ApEn), sample entropy (SampEn), and Lempel-Ziv complexity (LZ) are alternative tools for time series analysis for capturing nonlinear dynamics. In addition, scholars applied these entropies to several fields in econophysics [32,33,34,35,36] and financial phenomena [37,38,39,40,41,42]. Therefore, in this paper, we apply these three metrics to the price sequence of the cryptocurrency market for investigating the evolutionary unpredictability or complexity. Rather than trusting only one algorithm, it seems important to cross-check with several algorithms. In addition, using the sliding time window analysis, we observed how the complexity of the cryptocurrency market changes with time and how it changes with events such as the COVID-19 pandemic and vaccination. Although COVID-19 is still undergoing, the findings of this study can help related agents and global policymakers develop the right decisions for the short, mid, and long-term, ultimately contributing to reducing market uncertainty.

The remainder of this study is organized as follows: Section 2 introduces three general complexity algorithms on the basis of nonlinear metrics. Section 3 presents the results of the complexity of the cryptocurrency market from the bull to the current COVID-19 pandemic outbreak. Finally, Section 4 summarizes our conclusions and introduces future research directions.

## 2. Materials and Methods

### 2.1. Approximate Entropy (ApEn)

Approximate Entropy (ApEn) [28] measures sequential and temporal regularity and irregularity of a nonlinear time series. We consider a time sequence $S_{i}=\left[x\left(i\right),x\left(i+1\right),\cdots ,x\left(i+m-1\right)\right]$, where i=1,2,⋯,N−m+1, *N* is the length of the sequence, and *m* denotes the embedding dimension (m≥1). We describe the general process of estimating ApEn [28,43] and express the distance between $S_{i}$ with others as follows:

(1) $$d\left[S_{i},S_{j}\right]=\max_{k=0,1,\cdots ,m-1}\left|x(i+k)-x(j+k)\right|.$$

Let E[x(n)] represent the average of the time value x(n) and θ as the threshold of distance $d[S_{i},S_{j}]$, and we calculate as follows:

(2) $$\theta =\alpha \sqrt{E\left[x{\left(n\right)}^{2}\right]-[E\left[x\left(n\right)\right]{]}^{2}}.$$

where α is a modified coefficient. Next, we count the amount of distance less than θ, which we describe as follows:

(3) $$N_{i}^{m}\left(\theta \right)=\underset{j=1,2,..,N-m+1,j\neq i}{num}\left\{d\left[S_{i},S_{j}\right]<\theta \right\},$$

Therefore, we compute the average self-correlation $C_{i}^{m}\left(\theta \right)$, which is the ratio of $N_{i}^{m}\left(\theta \right)$ to the total number of period N−m+1, as $N_{i}^{m}\left(\theta \right)/L$. Then, we calculate the correlation degree $\Phi^{m}\left(\theta \right)$ of all sequence values $S_{i}$ under the dimension *m* as follows:

(4) $$\Phi^{m}\left(\theta \right)=\frac{1}{N-m+1}\sum_{i=1}^{N-m+1}\ln C_{i}^{m}\left(\theta \right).$$

In the same way, when the embedding dimension increases to m+1, we calculate the correlation degree $\Phi^{m+1}\left(\theta \right)$ according to the equations above. Finally, we estimate the ApEn of the time sequence as follows:

(5) $$ApEn\left(m,\theta ,N\right)=\Phi^{m}\left(\theta \right)-\Phi^{m+1}\left(\theta \right).$$

### 2.2. Sample Entropy (SampEn)

Sample entropy (SampEn) [30] measures the randomness of a sequence of data. It is also similar to ApEn and belongs to a family of many other variations [44,45]. SampEn refers to the negative logarithm of conditional probability between two sequences within a tolerance θ, excluding self-matches. For a given time sequence $S_{i}=\left[x\left(i\right),x\left(i+1\right),\cdots ,x\left(i+N-1\right)\right]$ denotes the length of *N*, we calculate SampEn as follows. The Chebyshev distance between $S_{i}$ and $S_{i+1}$ is the same as equation (1) of ApEn. For a given $S_{i}$ and $S_{j}$, we count the number of *j* (1≤j≤N−m+1,j≠i), $B_{i}\left(\theta \right)$, such that the distance is less than or equal to a threshold θ.

(6) $$B_{i}^{m}\left(\theta \right)=\frac{B_{i}\left(\theta \right)}{N-m},B^{m}\left(\theta \right)=\frac{1}{N-m+1}\sum_{i=1}^{N-m+1}B_{i}^{m}\left(\theta \right).$$

If we repeat this procedure by increasing the dimension to m+1, then we can calculate $B_{i}^{m+1}\left(\theta \right)$ and $B^{m+1}\left(\theta \right)$. Thus, they are the probabilities that would match for *m* and m+1 samples, respectively. Finally, we denote SampEn as follows:

(7) $$SampEn\left(m,\theta \right)=\lim_{N\to \infty }\left\{-\ln\left[\frac{B^{m+1}\left(\theta \right)}{B^{m}\left(\theta \right)}\right]\right\}.$$

For a finite time sequence, we can calculate the above equation by using the statistic:

(8) $$SampEn\left(m,\theta ,N\right)=-\ln\left[\frac{B^{m+1}\left(\theta \right)}{B^{m}\left(\theta \right)}\right].$$

Hence, a large value of ApEn and SampEn denotes strong irregularity and unpredictability of the time sequence.

### 2.3. Lempel-Ziv Complexity (LZ)

Lempel-Ziv complexity (LZ) [31] is an efficient measure for the complexity of a time sequence. LZ complexity uses the self-delimiting learning process as a basis. It combines the complexity with new patterns recursively. Before we calculate this metric, we should transform the time sequence into a binary symbolic sequence by comparing it with the median value $Med(S_{i})$ of the symbol sequence $S_{i}=\left\{s\left(i\right)|i=1,2,\cdots ,N\right\}$ using the following equation:

(9) $$s\left(i\right)=\left\{\begin{array}{cc} 0, & if\;x\left(i\right)<Med\left(S_{i}\right)\\ 1, & otherwise \end{array}\right.$$

The median is chosen due to its robustness to value within the time sequences [46,47]. LZ complexity consists of two primary operations: reproduction and production [48]. After obtaining the transformed sequence $S_{i}=\left\{s\left(i\right)|i=1,2,\cdots ,N\right\}$, we initialize R=s(1) and Q=s(2) and the complexity index c(N)=1.

Then, we combine *R* with *Q* as RQ, and $RQ_{L}$ denotes the sub-string obtained from RQ via deleting the last one at RQ. If *Q* belongs to the sub-sequence of $RQ_{L}$, we update *Q* by adding the next value and repeat the process of reproduction until *Q* has contained the last value of *R*. Otherwise, *Q* is a newly shown pattern in a sequence. Let *R* as RQ and renew *Q* with the following value to update RQ and $RQ_{L}$. This process increases the complexity index. We repeat all of the previous processes until the end of the sequence and then use c(N) to obtain (2≤c(N)≤N). However, we can only calculate the upper bound of all patterns for a finite sequence L(N):

(10) $$L\left(N\right)=\frac{N}{\ln(N)+1}.$$

The normalized index of complexity LZ is as follows:

(11) $$LZ=\frac{c(N)}{L(N)}.$$

When *N* increases, we can simplify LZ as follows:

(12) $$LZ=\frac{c(N)\ln(N)}{N}.$$

Hence, a large value of LZ implies strong complexity with new patterns.

## 3. Results

### 3.1. Data

According to Yahoo Finance, in November 2020, approximately 367 cryptocurrencies are being traded. To analyze the growth of cryptocurrency to the COVID-19 pandemic, we used a period of approximately four years from January 2017 to July 2021. We excluded the recently developed markets and those with discontinued trading now. We obtained price data in 43 out of 367 cryptocurrencies with 1692 data points for each. Table 1 presents the list of 43 cryptocurrency markets in descending order from the largest market cap.

**Figure 1:**
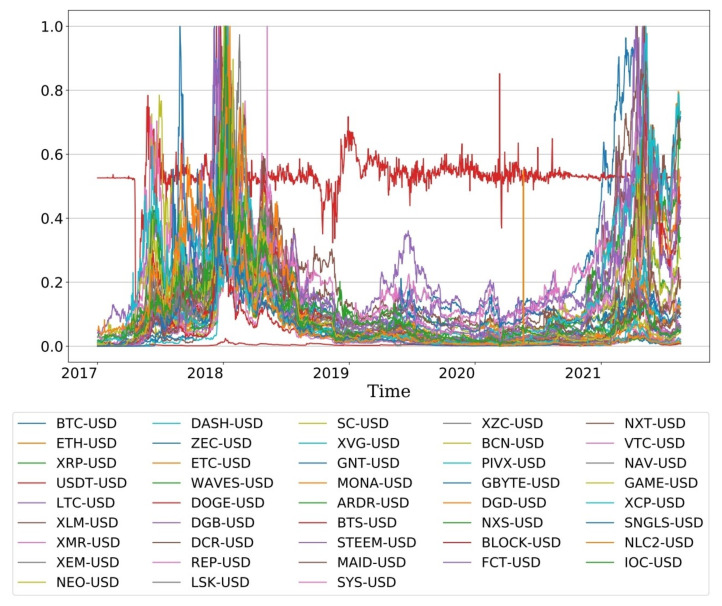
Scaled plot of 43 cryptocurrency markets from the bull market in 2017 to the recent COVID-19 pandemic.

Figure 1 presents the evolution of prices from 2017 when the cryptocurrency was growing to the recent pandemic. To compare the similarity patterns of the data, we scaled the prices of all markets equally between 0 and 1. As you can see, the dynamics of the market appear differently according to each period. In terms of trend, overall prices rose sharply in 2017 and 2018, and although there was often a move to increase in 2019 and 2020, it was limited by COVID-19. The trend of the cryptocurrency market shows that prices rose sharply in 2017 and 2018, followed by a stagnant move until the recent COVID-19. Again from 2020, prices are rising sharply with COVID-19. Therefore, it is necessary to explore the complexity of the cryptocurrency market through the entropy evolution in order to understand the rapid fluctuations from the past with small economic risks to the COVID-19 pandemic.

### 3.2. Complexity Evolution by Time and Market Cap

**Figure 2:**
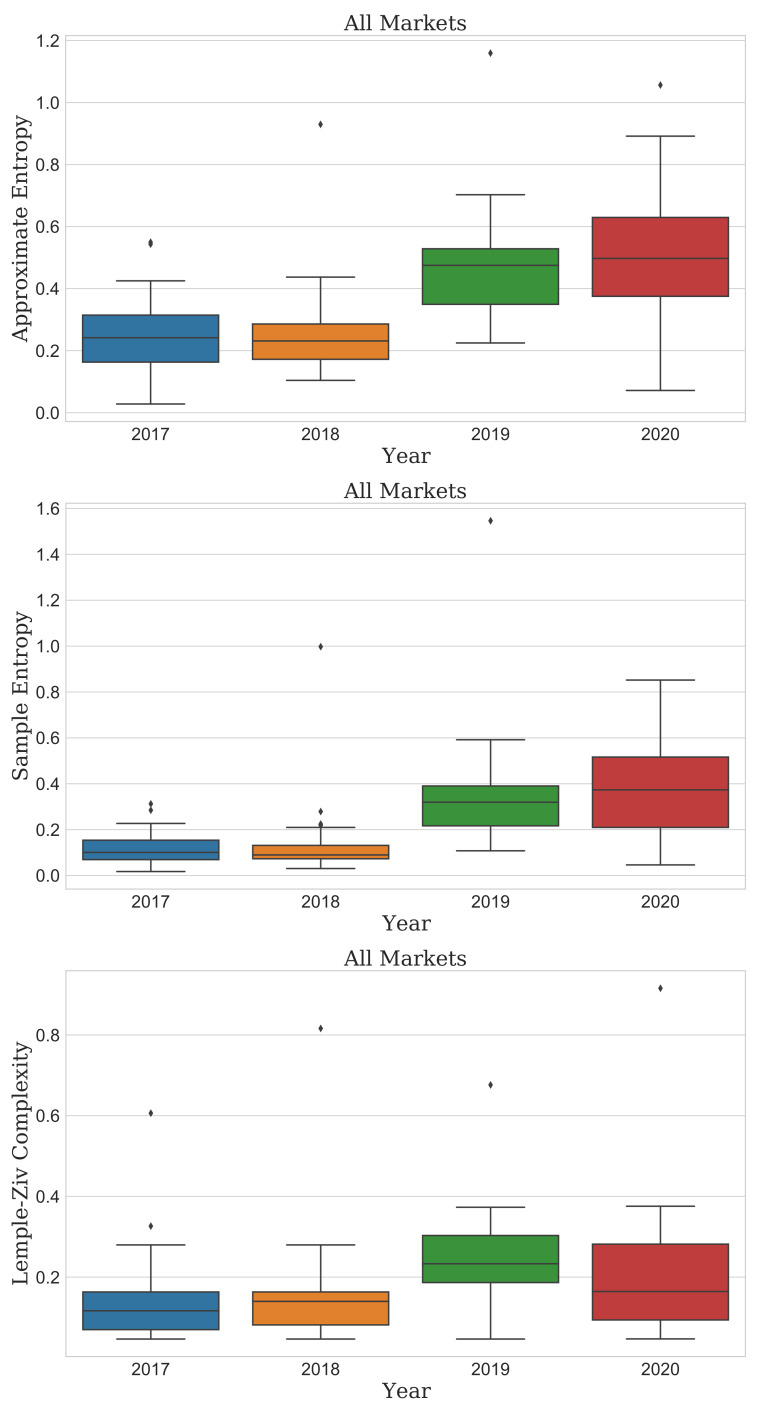
Boxplots of the ApEn, SampEn, and LZ for the cryptocurrency markets from the bull to current COVID-19 pandemic. Each year includes 43 markets. Diamond symbols are outliers.

Figure 2 presents the results of the three complexity algorithms. ApEn and SampEn gradually increased until the recent COVID-19 pandemic outbreak. The LZ complexity distribution increased as it widened in 2019∼2020 more than in 2017∼2018. The median in 2019 was the highest in the entire period. In descriptive statistics, the interquartile range (IQR) is a measure of statistical dispersion and a widely accepted robust measure of scale. The IQR is equal to the difference between 75th and 25th (Q1) percentiles, and most of the values for each year were within the 1.5 × IQR. However, this situation did not hold for some values, so they were not a completely normal distribution.

**Figure 3:**
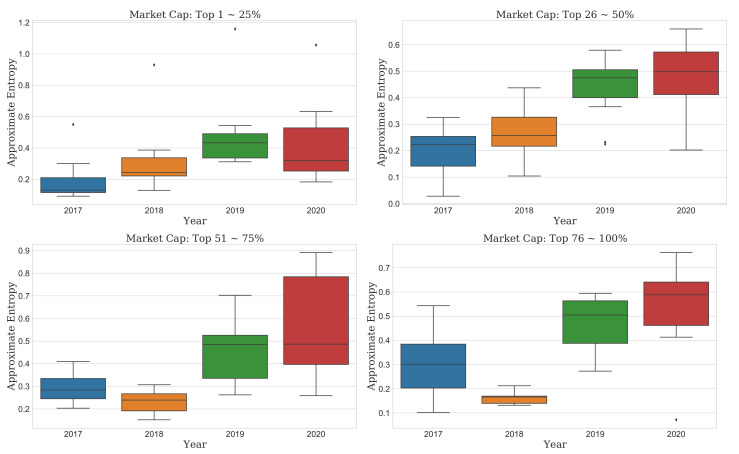
Boxplots of the ApEn for each market cap from the bull to current COVID-19 pandemic year. Each group includes 11 markets approximately.

**Figure 4:**
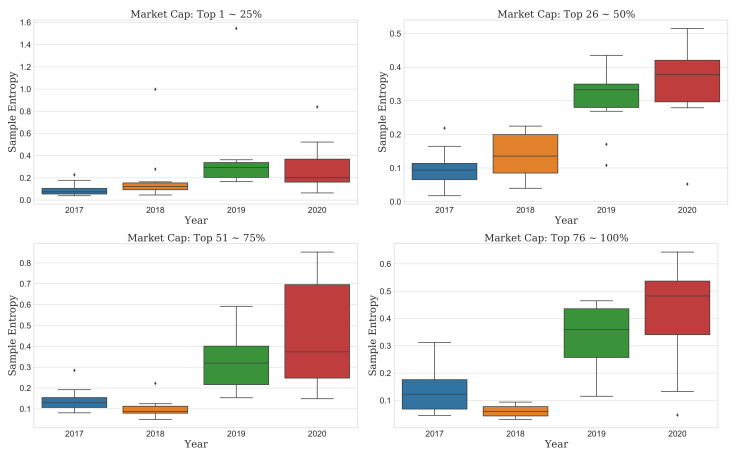
Boxplots of the SampEn for each market cap from the bull to current COVID-19 pandemic year. Each group includes 11 markets approximately.

**Figure 5:**
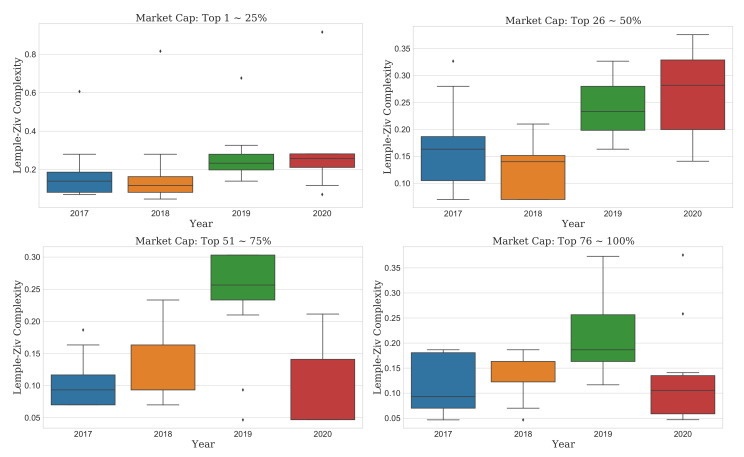
Boxplots of the LZ for each market cap from the bull to current COVID-19 pandemic year. Each group includes 11 markets approximately.

This finding means that the recent COVID-19 pandemic could make market predictions more difficult than in the past. Thus, we should consider it in detail. As the cryptocurrency market is highly volatile and newly emerged, the change in complexity depending on the market cap might be different from the result of the overall market. The unpredictability of the entire market should not be generalized to each market. Accordingly, we further analyzed the evolution of complexity by the market cap, as shown in Figure 2, Figure 3, Figure 4 and Figure 5.

First, the distribution of ApEn in all cryptocurrency markets decreased from 2017 to 2018 on a 75% quantile basis, but it continuously increased until the current COVID-19 pandemic year (top of Figure 2). However, in market cap the top “1–50%”, the complexity has been continuously increasing from 2017 to 2020, and we observed no particular decrease. It also was confirmed that the complexity/unpredictability of the cryptocurrencies with a market cap of “51–100%” was very low in 2018 compared to other years. The distribution of ApEn has significantly increased in 2019 and 2020 during the COVID-19 pandemic compared to 2017 and 2018). Furthermore, in particular, it shows that complexity/unpredictability increased more in 2020 than in 2019 in each market cap result. In 2020, the complexity of the “51–75%” market cap has the widest range of IQR, so it shows that cryptocurrency with a very diverse complexity is distributed due to the COVID-19 pandemic in market cap being the top “51–75%”. (bottom left of Figure 3).

Second, the SampEn results did not differ from the ApEn results in all cryptocurrency markets. (middle of Figure 2). Furthermore, the SampEn results and ApEn results were similar even after looking at the detailed results of the complexity of the cryptocurrency market according to the market cap. The difference between the result of SampEn and the result of ApEn can be confirmed in that the IQR of SampEn has a narrower result than that of ApEn. That is, it showed that the density of complexity of the cryptocurrencies appeared more in SampEn than ApEn. (Figure 4).

Finally, LZ complexity required another accurate interpretation. In this metric, all markets had shown the most unpredictability in 2019 and lower in 2020. Moreover, 2017 and 2018 were even lower and similar to each other (bottom of Figure 2). Looking at the detailed results of LZ complexity according to the market cap of the cryptocurrency market, the difference in LZ complexity according to the market cap was very large.

The cryptocurrencies with a market cap of the top “1–25%” showed little difference in LZ complexity by year. The cryptocurrencies in the top “26–50%” have the lowest LZ complexity value in 2018 but show an increasing trend from 2017 to 2020. However, in the top “51–100%” of cryptocurrencies, the LZ complexity increased significantly in 2019, and the result in 2020 is similar to 2017 and 2018 again. Therefore, the COVID-19 pandemic affected the entire market, especially the cryptocurrencies with a market cap of the top “51–100%” (Figure 5). However, we should still consider the effect of the current health crisis on each market cap, given the increasing complexity. Among the market caps, the top “26–50%” group had the highest range, so this market cap seems to have been most affected by the COVID-19 pandemic.

The market price had changed in various ways with the market complexity and uncertainty. We showed that complexity in the whole market and complexity by the market cap is different. Therefore, this result should be interpreted closely under the market cap.

### 3.3. Comparison Test of Complexity Evolution

We performed a comparison test on the distribution of complexity metrics. After sorting the market cap in descending order, we divided it into four groups and then applied three comparison tests. In Figure 3, Figure 4 and Figure 5, the distributions were slightly out of a normal distribution, so we applied the Mann–Whitney U test (the test for equality of means between two samples), Kruskal–Wallis test (the test for equal means among more than three samples), and Levene test (the test for equal variances among more than three samples) to the distribution of all metrics. Table 2 and Table 3 show the results of the top two groups “1–50%” and the bottom two groups “51–100%”, respectively. We conducted a total of three statistical comparisons as follows: first, we conduct two-sample comparisons for 2020, with each from 2017 to 2019. Then, we determined whether the metric mean and variance of all years were the same or not. Finally, for each market cap, we checked whether the increasing or decreasing pattern from 2017 to 2020 was statistically similar or not. We used a statistical significance level of 10%.

According to ApEn of Table 2a, our findings failed to support the null hypothesis (same mean), confirming that the means were different. However, given that its variance accepts the null hypothesis (same variance), we observed no year-to-year difference. For each year’s comparison, the null hypotheses of less than (left > right) between 2020 and each of 2017 and 2018 are rejected. The current health crisis affected 2020 the most, and the complexity had increased. Moreover, the results of SampEn and LZ complexity were similar to those of ApEn. Therefore, the average of metrics had increased until the COVID-19 pandemic outbreak. Following the metrics in Table 2b, our findings rejected the null hypothesis in the same direction without any difference in the algorithm. Therefore, the evolutionary effect of the market cap top “26–50%” is the largest in 2020.

However, the result of the market cap top “51–100%” is different from the result of the top “1–50%”. First, it rejects the null hypothesis (same mean) in all metrics, showing that the mean for all years is different. In addition, ApEn and SampEn reject the null hypothesis (same variance) and show that the variances for all years are different. That is, the market cap top “51–100%” shows a larger difference in both the mean and variance than the top “1–50%” and is highly volatile. For each year’s comparison in the SampEn and ApEn entropy, the null hypotheses of less than (left > right) between 2020 and each of 2017 and 2018 are rejected in the market cap top “51–75%”. However, those of less than (left > right) between 2020 and each of 2017, 2018, and 2019 are rejected in the top “76–100%”. That is, the top “76–100%” group is most affected by the COVID-19 pandemic. For each year’s comparison in the LZ, complexity is decreasing in 2020. This point is different from the result of the top “1–50%”. Therefore, the lower the market cap, the greater the volatility of complexity and the more COVID-19 has an effect. In other words, the higher the market cap, the higher the unpredictability during the COVID-19 pandemic period. In addition, combining these metrics with another metric in cross-examination was more helpful in decision-making than using only one metric.

### 3.4. Vaccination Effect on Complexity

Previously, it was confirmed through statistical comparison that complexity evolution significantly increased with time. However, it includes only data up to 2020, when the coronavirus occurred, and excludes 2021, when a vaccine is being developed and the global vaccination rate is increasing. The reason is that there are about 365 samples per year from 2017 to 2020, but there are 232 samples in 2021. Because 2021 is not over, the sample numbers for 2021 are different from other years. If there is a difference in the number of samples, a problem may arise when comparing by year. There will be a problem with the reliability of time evolution and the robustness of statistics. To solve this problem, the sliding time window analysis [20] is necessary to analyze the changes of the dynamic behavior of complexity in the cryptocurrency market from 2017 to 2021 to quantitatively compare the complexity of 2021 when the vaccination rate is increasing and mutations occur. For robustness of entropy, the time window is set to 1 quarter (90 days). Moreover, the results of the COVID-19 test have a weekly trend because vaccine tests are mainly conducted on weekdays. Therefore, a time interval is set to 2 weeks (14 days) for stable calculation that is not affected by the weekly trend.

**Figure 6:**
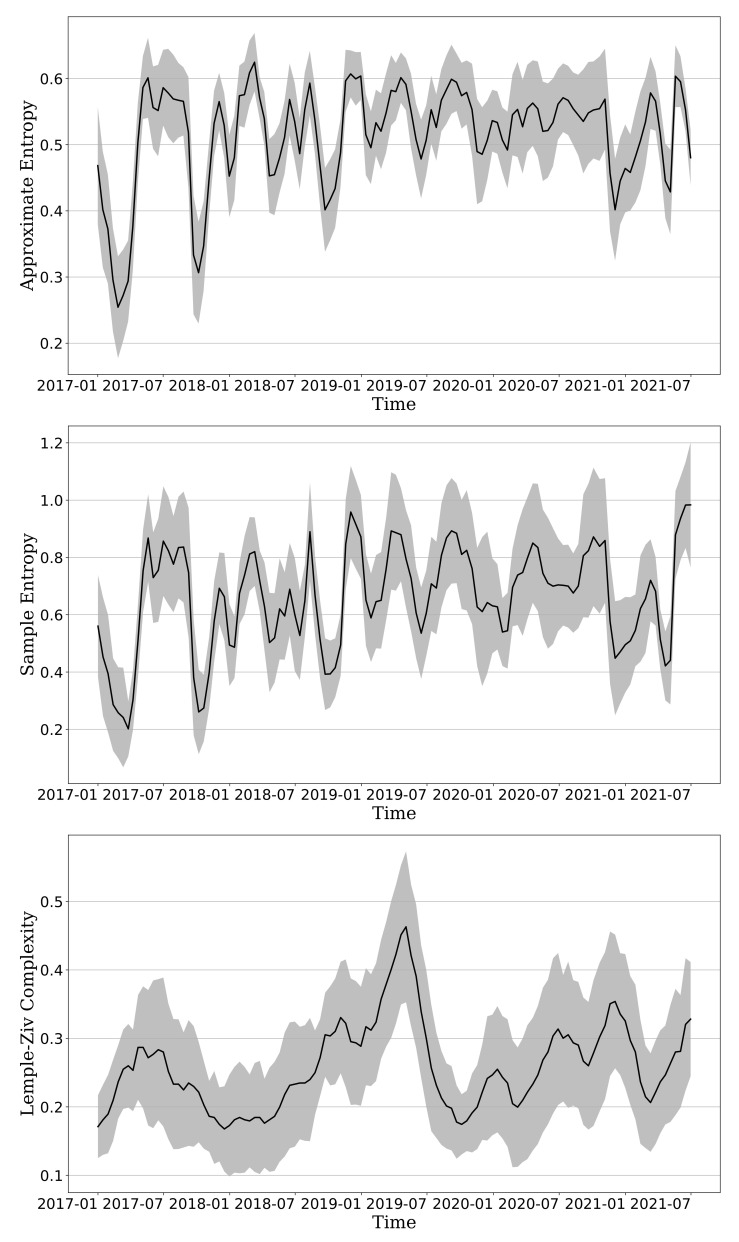
Time evolution of ApEn, SampEn, and LZ. The black line is the average value of 43 cryptocurrencies in market, and the gray shade is the 95% confidence interval.

Figure 6 shows the time evolution of three complexity measures over time. It shows the trend of complexity in the cryptocurrency market. In the case of ApEn, it shows the lowest point in 2017, but the ApEn value is gradually increasing, and it is maintained at a high level in 2020 during the COVID-19 outbreak. The overall volatility has increased even though a vaccine was developed in 2021. SampEn is similar to ApEn, but after 2020, it has been slightly different. With the advent of COVID-19, it has always had a high value, but with the news of vaccine development, the unpredictability seemed to decrease. However, in 2021, the value of SampEn starts to increase again, showing the highest unpredictability in history in July. The volatility is larger than that of ApEn. Lastly, the LZ complexity showed the highest value in the middle of 2019 but has decreased since the advent of COVID-19 and has been a steady upward trend until now. In summary, the cryptocurrency market has recently increased in complexity and thus unpredictability is increasing. Although the complexity of the cryptocurrency market has temporarily decreased due to the development of a vaccine, the complexity of the cryptocurrency market has still been increasing since then. Vaccination rates are rising worldwide, but it is estimated that many other factors, such as the emergence of the delta virus or foreshadowing of tapering, are adding to the complexity of the cryptocurrency market.

## 4. Discussion

After the financial crisis in 2008 that put the global economy into a panic, we are currently experiencing a new pattern of global economic panic due to the COVID-19 pandemic. Likewise, the cryptocurrency market has also advanced since the financial crisis in 2008 and many cryptocurrencies have emerged and disappeared due to trading issues. From an investment perspective, we investigate the unpredictability of the cryptocurrency from 2017 to the present COVID-19 pandemic.

To confirm the evolutionary complexity, we use three general complexity analyses on the basis of nonlinear measures: Approximate Entropy, Sample Entropy, and Lempel-Ziv complexity. By applying these measures to the price sequence of 43 markets, we investigate the market unpredictability and newly generated patterns. We also describe the market characteristics in detail and conduct comparison tests in accordance with the market caps. The market prices could have recorded the highest number in 2017 and 2018. However, the actual complexity has increased in the COVID-19 pandemic, resulting in more unpredictable patterns. All complexity metrics of all markets showed different patterns, so we could not determine that the unpredictability was affected by the COVID-19 pandemic outbreak alone. Given the outliers exceeding the 1.5 × IQR of distribution, the complexity or unpredictability in all markets might be different from that of each market cap size. The results of the three non-parametric tests are suitable for non-normal distribution comparisons, and the average of metrics has increased until the COVID-19 pandemic. ApEn and SampEn reject the null hypothesis in the same way regardless of the market cap. LZ complexity is useful in interpreting the evolution of complexity because it quantitatively and differently rejected the null hypothesis for each market cap. Furthermore, in the case of ApEn and SampEn, the complexity showed a steady increase from 2017 to 2020. In particular, 2019 and 2020, which were affected by COVID-19, were high, and 2020 was the highest. However, in the case of LZ complexity, 2019 and 2020 were high too, but in particular, 2019 was the highest. Combining LZ complexity with other metrics in cross-examination is more helpful than using only one metric. Finally, using the sliding time window analysis, we observed the change in the complexity of the cryptocurrency market according to events. Although the complexity of the cryptocurrency market temporarily decreased due to vaccination, it was confirmed that the trend of complexity was rising again.

Our study of the complexity of the cryptocurrency market could be significant information in predicting the future of the market using big data and AI, which is becoming important. Therefore, in the future, we aim to investigate the predictive performance of the cryptocurrency market and develop methods to improve it.

## Figures and Tables

**Table 1:** List of cryptocurrency markets by the market cap.

Market Cap	Name	Abbreviation	Market Cap	Name	Abbreviation	Market Cap	Name	Abbreviation	Market Cap	Name	Abbreviation
1	Bitcoin	BTC-USD	12	EthereumClassic	ETC-USD	23	Ardor	ARDR-USD	34	Blocknet	BLOCK-USD
2	Ethereum	ETH-USD	13	Waves	WAVES-USD	24	BitShares	BTS-USD	35	Factom	FCT-USD
3	XRP	XRP-USD	14	Dogecoin	DOGE-USD	25	Steem	STEEM-USD	36	Nxt	NXT-USD
4	Tether	USDT-USD	15	DigiByte	DGB-USD	26	MaidSafeCoin	MAID-USD	37	Vertcoin	VTC-USD
5	Litecoin	LTC-USD	16	Decred	DCR-USD	27	Syscoin	SYS-USD	38	NavCoin	NAV-USD
6	Stellar	XLM-USD	17	Augur	REP-USD	28	Zcoin	XZC-USD	39	GameCredits	GAME-USD
7	Monero	XMR-USD	18	Lisk	LSK-USD	29	Bytecoin	BCN-USD	40	Counterparty	XCP-USD
8	NEM	XEM-USD	19	Siacoin	SC-USD	30	PIVX	PIVX-USD	41	SingularDTV	SNGLS-USD
9	NEO	NEO-USD	20	Verge	XVG-USD	31	Obyte	GBYTE-USD	42	NoLimitCoin	NLC2-USD
10	Dash	DASH-USD	21	Golem	GNT-USD	32	DigixDAO	DGD-USD	43	IOCoin	IOC-USD
11	Zcash	ZEC-USD	22	MonaCoin	MONA-USD	33	Nexus	NXS-USD			

**Table 2:** Result of comparison test of complexity evolution by each market cap (Top 1–50%).

**(a) Market cap: Top “1–25%”**					
**Algorithm**	**Null Hypothesis**	**“2017 = 2020”**	**“2018 = 2020”**	**“2019 = 2020”**	**“2017 = 2018 = 2019 = 2020”**
Approximate Entropy	left = right	0.00 *	0.19	0.32	-
	left > right	0.00 *	0.09 *	0.85	-
	left < right	1.00	0.92	0.16	-
	same mean	-	-	-	0.00 *
	same variance	-	-	-	0.66
Sample Entropy	left = right	0.00 *	0.05 *	0.43	-
	left > right	0.00 *	0.02 *	0.80	-
	left < right	1.00	0.98	0.22	-
	same mean	-	-	-	0.00 *
	same variance	-	-	-	0.59
Lemple-Ziv Complexity	left = right	0.04 *	0.03 *	0.60	-
	left > right	0.02 *	0.01 *	0.30	-
	left < right	0.98	0.99	0.72	-
	same mean	-	-	-	0.02 *
	same variance	-	-	-	0.98
**(b) Market cap: Top “26–50%”**					
**Algorithm**	**Null Hypothesis**	**“2017 = 2020”**	**“2018 = 2020”**	**“2019 = 2020”**	**“2017 = 2018 = 2019 = 2020”**
Approximate Entropy	left = right	0.00 *	0.00 *	0.36	-
	left > right	0.00 *	0.00 *	0.18	-
	left < right	1.00	1.00	0.84	-
	same mean	-	-	-	0.00 *
	same variance	-	-	-	0.85
Sample Entropy	left = right	0.00 *	0.00 *	0.26	-
	left > right	0.00 *	0.00 *	0.13	-
	left < right	1.00	1.00	0.88	-
	same mean	-	-	-	0.00 *
	same variance	-	-	-	0.43
Lemple-Ziv Complexity	left = right	0.00 *	0.00 *	0.21	-
	left > right	0.00 *	0.00 *	0.11	-
	left < right	1.00	1.00	0.91	-
	same mean	-	-	-	0.00 *
	same variance	-	-	-	0.51

Note that * denotes 10% level of significance.

**Table 3:** Result of comparison test of complexity evolution by each market cap (Top 51–100%).

**(a) Market cap: Top “51–75%”**					
**Algorithm**	**Null Hypothesis**	**“2017 = 2020”**	**“2018 = 2020”**	**“2019 = 2020”**	**“2017 = 2018 = 2019 = 2020”**
Approximate Entropy	left = right	0.00 *	0.00 *	0.32	-
	left > right	0.00 *	0.00 *	0.16	-
	left < right	1.00	1.00	0.85	-
	same mean	-	-	-	0.00 *
	same variance	-	-	-	0.00 *
Sample Entropy	left = right	0.00 *	0.00 *	0.26	-
	left > right	0.00 *	0.00 *	0.13	-
	left < right	1.00	1.00	0.88	-
	same mean	-	-	-	0.00 *
	same variance	-	-	-	0.00 *
Lemple-Ziv Complexity	left = right	0.32	0.16	0.00 *	-
	left > right	0.86	0.93	1.00	-
	left < right	0.16	0.08 *	0.00 *	-
	same mean	-	-	-	0.00 *
	same variance	-	-	-	0.72
**(b) Market cap: Top “76–100%”**					
**Algorithm**	**Null Hypothesis**	**“2017 = 2020”**	**“2018 = 2020”**	**“2019 = 2020”**	**“2017 = 2018 = 2019 = 2020”**
Approximate Entropy	left = right	0.01 *	0.00 *	0.10 *	-
	left > right	0.00 *	0.00 *	0.05 *	-
	left < right	1.00	1.00	0.96	-
	same mean	-	-	-	0.00 *
	same variance	-	-	-	0.08 *
Sample Entropy	left = right	0.01 *	0.00 *	0.12	-
	left > right	0.00 *	0.00 *	0.06 *	-
	left < right	1.00	1.00	0.95	-
	same mean	-	-	-	0.00 *
	same variance	-	-	-	0.02 *
Lemple-Ziv Complexity	left = right	0.79	0.34	0.03 *	-
	left > right	0.40	0.85	0.99	-
	left < right	0.63	0.17	0.02 *	-
	same mean	-	-	-	0.03 *
	same variance	-	-	-	0.50

Note that * denotes 10% level of significance.

## Data Availability

Not applicable.
